# Modification of 316L Stainless Steel, Nickel Titanium, and Cobalt Chromium Surfaces by Irreversible Immobilization of Fibronectin: Towards Improving the Coronary Stent Biocompatibility

**DOI:** 10.3390/molecules29204927

**Published:** 2024-10-18

**Authors:** Hesam Dadafarin, Evgeny Konkov, Hojatollah Vali, Irshad Ali, Sasha Omanovic

**Affiliations:** 1 Department of Chemical Engineering, McGill University, 3610 University St., Montreal, QC H3A 0C5, Canada; hesam.dadafarin@mail.mcgill.ca (H.D.); evgeny.konkov@mail.mcgill.ca (E.K.); 2 Department of Anatomy and Cell Biology, McGill University, 3640 University St., Montreal, QC H3A 0C7, Canada; hojatollah.vali@mcgill.ca; 3 Department of Chemical Engineering, Faculty of Mechanical, Chemical and Industrial Engineering, University of Engineering & Technology, Jamrud Road, Peshawar 25000, Pakistan; irshad.ali@mail.mcgill.ca

**Keywords:** biomaterials, monolayers, surface properties, electrochemical techniques, infrared spectroscopy (IR)

## Abstract

An extracellular matrix protein, fibronectin (Fn), was covalently immobilized on 316L stainless steel, L605 cobalt chromium (CoCr), and nickel titanium (NiTi) surfaces through an 11-mercaptoundecanoic acid (MUA) self-assembled monolayer (SAM) pre-formed on these surfaces. Polarization modulation infrared reflection adsorption spectroscopy (PM-IRRAS) confirmed the presence of Fn on the surfaces. The Fn monolayer attached to the SAM was found to be stable under fluid shear stress. Deconvolution of the Fn amide I band indicated that the secondary structure of Fn changes significantly upon immobilization to the SAM-functionalized metal substrate. Scanning electron microscopy and energy dispersive X-ray analysis revealed that the spacing between Fn molecules on a modified commercial stent surface is approximately 66 nm, which has been reported to be the most appropriate spacing for cell/surface interactions.

## 1. Introduction

Upon the introduction of a new biomedical device (e.g., a stent, dental implant, hip joint replacement, or tissue-engineered substrate) into a human body, cells start to interact with the surface of the implant [1]. Depending on the physico-chemical properties of the surface of the implant, various cell interactions can induce severe complications inside the body, which may necessitate further therapies or even surgical replacement of the implant. Therefore, significant attention must be given to the properties of the implant surface, as its interaction with the host tissue often determines the success or failure of an implant [1]. Due to this reason, a considerable amount of research and clinical studies have been focused on enhancing the biocompatibility of biomedical device surfaces. To enhance the interaction of the implants with living cells and to manage undesired reactions, one approach is to coat the device’s surface with bioactive molecules. These molecules are typically intended to modulate how the biomaterial’s surface interacts with a specific living environment [2].

In-depth knowledge of the nature of the interactions between cells and surfaces has aided researchers in developing new methods to mitigate implant incompatibility issues [3,4]. Human tissue cells actively interact with their environment, which is typically the extracellular matrix (ECM). The ECM is a complex network of various molecules including an interlocking mesh of fibrous glycoproteins and glycosaminoglycans [5]. Among the ECM proteins, fibronectin (Fn), a large glycoprotein, has an extensive impact on cell/surface interactions.

Fn is a heavy dimeric protein that has two subunits of similar lengths. These two subunits are linked by a disulfide bridge at the carboxy termini [6]. Depending on the literature source, each subunit’s weight is reported to be between 220 and 270 kDa, for a combined protein weight of 440 to 500 kDa [7]. The length of each chain is 60 to 70 nm, and their diameter is 2.5 nm [6]. Each subunit consists of three repeating, globular modules (12 Type I, 2 Type II, and 15 to 17 Type III), on which there are different, highly specific binding sites for various cells. Cells interact with these binding sites through the cells’ transmembrane adhesion receptors called integrins [5].

Depending on the Fn molecular conformation, it has been proven that Fn can modulate cell attachment [8], migration [9], differentiation [10], proliferation [10], spreading [11,12], and wound healing [13] by triggering different intracellular signals.

Fibronectin can be immobilized on biomaterial surfaces using various techniques such as physical adsorption, covalent bonding, and layer-by-layer assembly [14,15,16]. Covalent immobilization, in particular, provides a stable attachment that resists desorption in physiological environments [17]. This method involves the activation of surface functional groups followed by the coupling of fibronectin, which preserves its bioactivity and enhances cell adhesion [18]. Recent studies have demonstrated that fibronectin-coated surfaces not only improve initial cell attachment but also promote long-term cellular functions, which are critical for successful tissue integration and healing [15,19]. Consequently, many researchers have investigated the possibility of using Fn to enhance the biocompatibility of medical devices [4,14,15,16]. The main idea is to pre-coat the surfaces of medical devices with Fn in order to minimize direct cell/surface interactions, which often cause incompatibility issues. The influence of surface Fn coating on the enhancement of the biocompatibility of various materials, such as gold [8], polystyrene [10], silicon [20], polyethylene glycol (PEG) [21], and 316L SS [22,23,24,25], has already been documented. In these studies, it has been determined that the complex structure of Fn has a remarkable impact on the modulation of cell attachment and subsequent cell responses. Based on the physicochemical properties of the underlying surface, such as charge, chemistry, and roughness, the Fn surface conformation can change [26], which can potentially result in different biological outcomes of the cell/stent surface interactions. For example, our previous study has shown that Fn adopts an open conformation on a negatively charged surface, while on a positively charged surface, the molecule is ‘closed’ [26]. The former conformation of Fn was found to interact more favorably with endothelial cells than the latter one. However, in both cases, the Fn-coated surface was shown to be a better substrate for cell/surface interactions than the naked (Fn-free) surface.

Of particular interest to the current study is addressing the major complication occurring after the implantation of cardiovascular stents, the so-called in-stent restenosis (ISR). IRS refers to a condition resulting from re-constriction of the artery at the stent location due to uncontrolled cell growth, more specifically that of smooth muscle cells (SMCs) [27]. In a normal artery, a monolayer of endothelial cells (ECs) lining the innermost layer of the vessel (intima) controls the proliferation of SMCs. During stent implantation, the intima is mechanically damaged by the implant and is thereby denuded of the EC monolayer; in consequence, the SMCs, which are located underneath the EC monolayer, i.e., in the middle layer of the vessel (media), become exposed to circulating blood. Exacerbated by the loss of the EC monolayer’s mediating effect, this exposure causes SMCs to proliferate rapidly, thus resulting in in-stent restenosis and impeded blood flow, on average, within six months of implantation [28].

There are several methods that have been employed to minimize the occurrence of ISR in patients [29]. One of the approaches is to use so-called drug-eluting stents (DESs) containing anti-proliferative and anti-inflammatory drugs that inhibit the proliferation of SMCs in the arterial lumen and decrease the inflammation of the artery, thus reducing the restenosis [30,31,32]. Although DESs reduce the incidence of restenosis, they may increase risks for late complications as compared to bare metal stents [33,34,35].

The ultimate goal of this study is to modify a stent surface with an irreversibly immobilized monolayer of Fn to attract ECs from the blood and facilitate their attachment to the stent surface [36]. Recent studies have demonstrated that fibronectin-coated surfaces not only improve initial cell attachment but also promote long-term cellular functions, which are critical for successful tissue integration and healing [15,19,37,38,39]. Additionally, it is well believed that the presence of ECM proteins, particularly Fn, can accelerate the migration of adjacent ECs to surfaces having a high concentration of immobilized Fn. Hence, in this study, a method developed in our laboratory [40] is applied to first form an 11-mercaptoundecanoic acid (MUA) monolayer on the surface of commonly used stent materials—316L stainless steel, nickel-titanium alloy (NiTi), and cobalt-chromium (L605)—followed by the covalent binding of Fn to this layer. In contrast to the simple physisorption of Fn on these metal surfaces, which is commonly performed in many laboratories, covalent binding reduces the risk of its detachment and subsequent replacement by other undesired proteins. Namely, if Fn attached to an implant surface is replaced by fibrinogen (Fg), an abundant ECM protein in blood, blood clotting may be triggered, which would potentially put the patient’s life in danger [41]. The stability of the attached Fn monolayer, its secondary structure, and surface coverage are also investigated in this study, all having a major impact on subsequent cell/surface interactions [42], which will be discussed in our upcoming publication.

## 2. Results and Discussion

### 2.1. Immobilization of Fn on MUA-Modified 316L, NiCi, and CoCr Surfaces

**Scheme 1:**
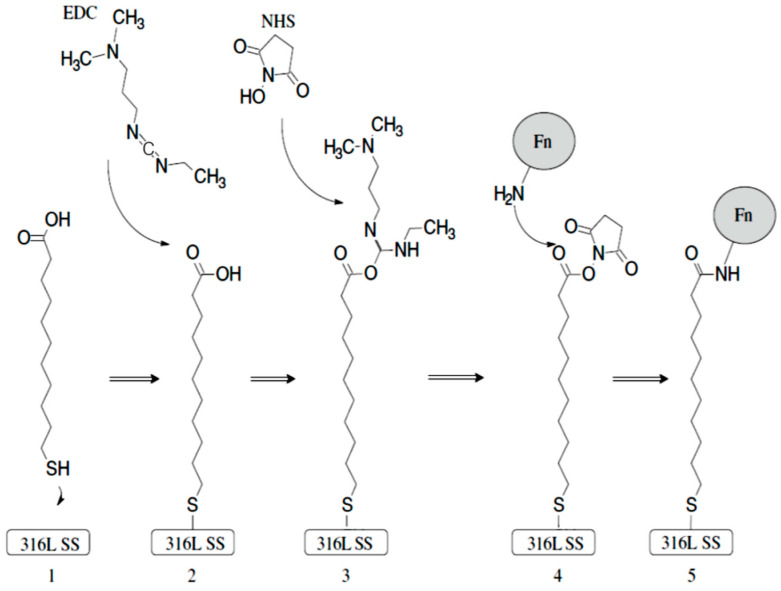
Covalent immobilization of fibronectin (Fn) onto a 316L stainless steel surface. (1) MUA film formation, (2) formation of COOH-EDC activated complex, (3) replacement of EDC by NHS to form an NHS-terminated film, (4) replacement of NHS with Fn, and (5) final structure of an Fn-modified 316L-SS surface [22].

Although 316L SS is still the material of choice to make coronary stents, L605 CoCr and Nitinol (NiTi) alloys are also increasingly used for the same purpose [1,24,25,43,44,45,46,47,48]. Therefore, it was interesting to investigate whether the electrochemical method used to form an MUA monolayer on a 316L SS surface presented in our previous paper [49] could also be used to form MUA monolayers on the latter two materials and subsequently to covalently attach Fn to the MUA monolayer, as shown in Scheme 1.

**Figure 1:**
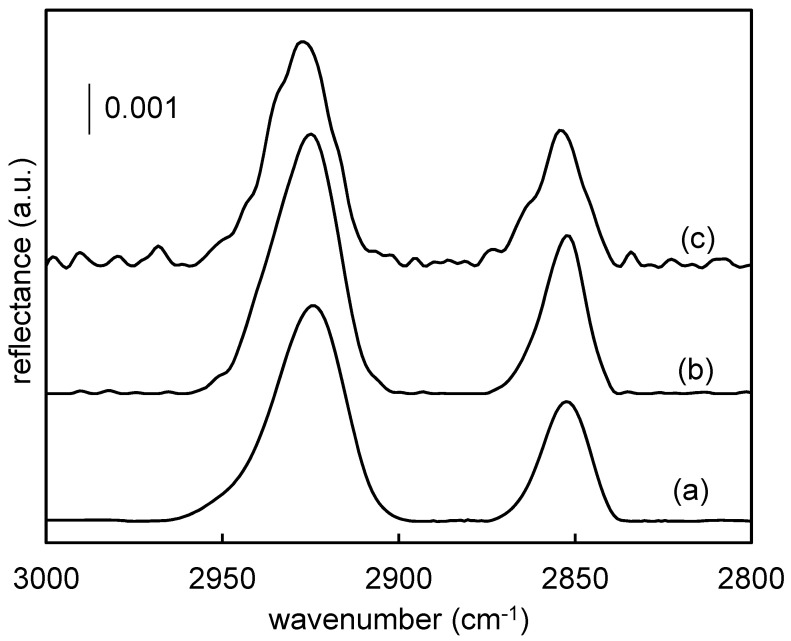
PM-IRRAS spectra of MUA SAM formed on (a) a 316L SS surface, (b) a CoCr surface, and (c) a NiTi surface.

The formation of an MUA monolayer on CoCr and NiTi surfaces was carried out according to the procedure presented in [49], to which the reader is invited to refer for further details. After electrochemically binding MUA to the samples and sonicating the samples in water and ethanol for 10 min to remove loosely bound MUA, the PM-IRASS response of the MUA on the 316L SS, CoCr, and NiTi surfaces was recorded and presented in Figure 1. A highly ordered, densely packed long-chain alkanethiol monolayer can be characterized by an asymmetric methylene stretching absorbance of ≤2918 cm^−1^ [50]. The peak positions corresponding to the asymmetric methylene stretching of MUA on 316L SS, CoCr, and NiTi (Figure 1) were found to be 2924 cm^−1^*,* 2925 cm^−1^, and 2926 cm^−1^, respectively. Thereby, these results indicate that a partially disordered MUA monolayer was formed on the surfaces. The other peaks recorded at 2851 cm^−1^ (curves a and b) and 2852 cm^−1^ (curve b) correspond to the symmetric methylene stretching of MUA on 316L SS, CoCr, and NiTi, respectively, and also confirm that the layer is partially disordered, since a perfectly ordered layer is characterized by a peak position at ≤2850 cm^−1^.

**Figure 2:**
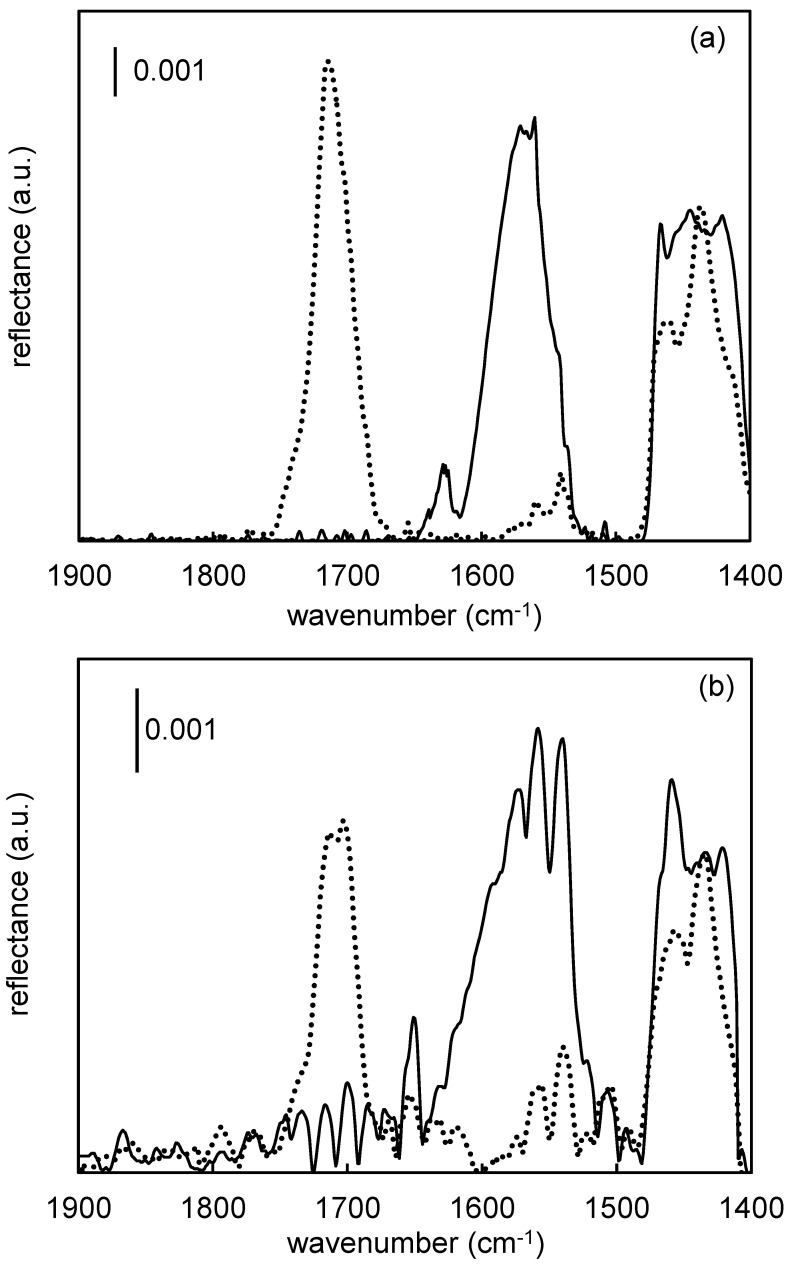
Lower wavenumber region of the PM-IRRAS spectra of MUA SAM formed on (**a**) a CoCr and (**b**) a NiTi surface: the solid line depicts the MUA response immediately after its formation and rinsing, whereas the dotted line represents the MUA response after treatment in a phosphoric acid solution.

**Figure 3:**
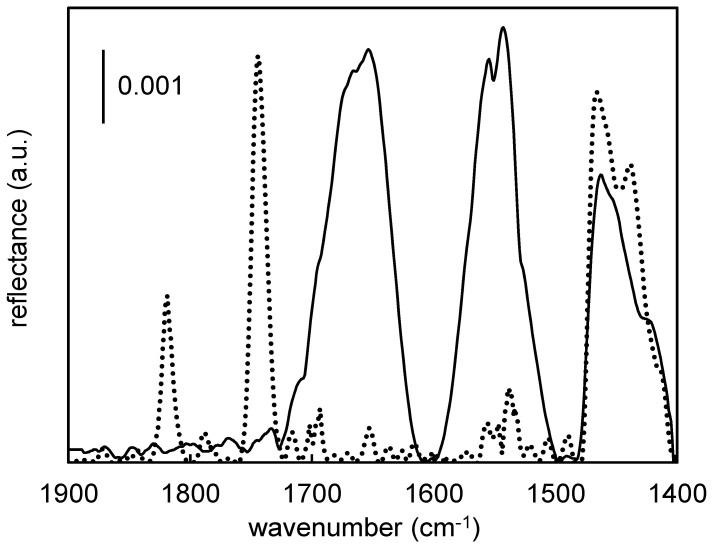
PM-IRASS responses of a modified 316L SS surface. The dotted line represents the response of an NHS activated surface, whereas the solid line represents that of an Fn-modified surface. Similar spectra were recorded with modified CoCr and NiTi surfaces (see Figure S1 in the Supplemental).

Figure 2 shows the infrared response of the MUA SAM on CoCr and NiTi surfaces in the lower wavenumber region, which depicts the spectra associated with the MUA carboxylate and methylene groups; a similar graph recorded with an MUA-enhanced 316L SS surface is presented in Figure 3 in our previous paper [49] and is thus not discussed here. The composite peaks in the regions of approximately 1650–1500 cm^−1^ and 1480–1400 cm^−1^ (solid line) represent asymmetric and symmetric vibrations of a deprotonated carboxylate terminal group of the MUA monolayer [51]. Our subsequent experiments on the chemical modification of MUA monolayers formed on CoCr and NiTi surfaces with an NHS intermediate revealed that the initially formed MUA monolayer (solid line) was not chemically active, which was due to the formation of a complex between the MUA terminal carboxylate group and the Na^+^ present in the MUA precursor electrolyte [49]. Thus, in order to destroy the complex and shift the equilibrium towards the formation of –COOH, MUA-modified CoCr and NiTi samples were immersed in a phosphoric acid solution (pH = 1.5). Indeed, the composite IR peak initially recorded in the 1650–1500 cm^−1^ region (solid line) disappeared, and a new peak at 1720 cm^−1^ appeared (dotted line). This peak corresponds to the C=O stretching of the free –COOH group [52], which confirms the destruction of the –COO-Na complex. The same effect was observed with the MUA bound on a 316L SS surface [49].

As already mentioned and schematically shown in Scheme 1, after the MUA monolayer activation, the next step in binding Fn was the modification of the MUA carboxyl group with NHS (Scheme 1), which was accomplished by incubating MUA-modified 316L SS, CoCr, and NiTi samples in an aqueous solution containing 25 mM EDC and 75 mM NHS. In order to confirm the formation of the succinimidyl ester intermediate, IR spectra of the NHS-modified 316L SS, CoCr, and NiTi surfaces were recorded in the lower wavenumber region (Figure 3, dotted line). The IR spectra for both CoCr and NiTi were almost identical to the 316L SS spectra in Figure 3 and are thus not presented here. A sharp peak at 1746 cm^−1^ corresponds to the asymmetric stretching of the NHS carbonyl groups [53]. The small peak at 1820 cm^−1^ originates from the carbonyl stretching of an ester formed by the NHS-MUA bond. Thus, the IR spectrum in Figure 3 (dotted line) evidences the successful formation of a covalent bond between NHS and the carboxylic group of the MUA monolayer on 316L SS (and likewise on the CoCr and NiTi surfaces; see Figure S1) [54]. Further, the peaks at 1420 and 1465 cm^−1^ (Figure 3, dotted line) correspond to the symmetric C=O stretching of the deprotonated COO- terminal groups and C-H bending of the underlying MUA layer, respectively [55].

Upon the successful activation of the MUA monolayer with NHS, the samples were immersed in an Fn-containing phosphate buffer for 18 h, during which the Fn molecules chemically reacted with the NHS groups covalently bound to the surface (Scheme 1). The samples were then rinsed with deionized water, sonicated in NaOH for 10 min, and then rinsed again with deionized water to remove any loosely physisorbed Fn. Figure 3 (solid line) shows the IR response of an Fn-modified 316L SS surface. Two peaks centered at 1655 cm^−1^ and 1545 cm^−1^ correspond to amide I and amide II bands, respectively, which confirm the presence of Fn on the surface [56]. In addition, the disappearance of the two sharp peaks at 1746 cm^−1^ and 1820 cm^−1^ belonging to NHS further confirms the substitution of NHS by Fn. Thereby, the result in Figure 3 (solid line) evidences the success of the procedure for the covalent immobilization of Fn on the 316L SS, NiTi, and CoCr surfaces. Furthermore, before collecting the PM-IRRAS spectra of immobilized Fn (Figure 3, solid line), all three sets of samples were sonicated in a highly alkaline chemical solution (0.1 M NaOH) for 10 min, which is known to desorb proteins from solid surfaces. Hence, the presence of amide bands in Figure 3 not only confirms the presence of Fn on the surface but also proves that the immobilized protein layer is resistant to harsh chemical conditions.

These findings are notably valuable because the proposed approach can be applied to modify the surfaces of at least three major alloys (316L SS, L605 CoCr, and NiTi) used to manufacture cardiovascular stents (and also other implants). As a foreseeable advantage of this surface enhancement technique, the selection of stent design material could potentially be based not chiefly on the biocompatibility of the original material but on its other important parameters, such as mechanical properties and price. This is because following the modification of the bare surface with Fn, the cells do not biologically “see” the alloy substrate surface but are more likely to interact with the outermost Fn layer, the properties of which do not depend on the substrate’s alloy composition.

Considering (i) the above, and since 316L SS is (ii) still a material of choice used to produce coronary stents, and (iii) it is less corrosion resistant than L605 CoCr and NiTi, in the remaining sections of this manuscript, the results obtained only with 316L SS are presented. However, one might expect to see at least similar results on L605 CoCr and NiTi.

### 2.2. Stability of the Fn Monolayer Covalently Bound to a 316L SS Surface

**Figure 4:**
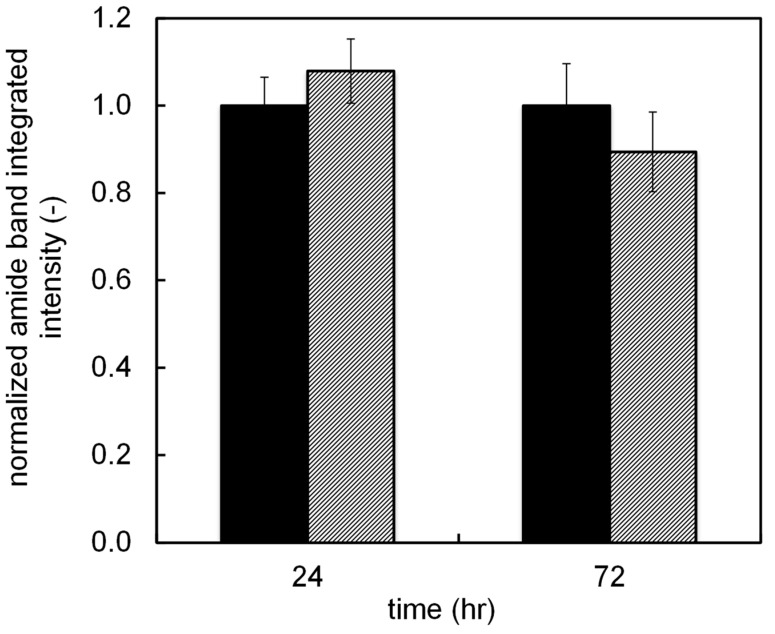
Time dependence of the normalized integrated intensity of the amide I vibration of a covalently immobilized Fn on an MUA-modified 316L SS surface before (solid bars) and after (patterned bars) exposure to simulated laminar flow (PBS, pH = 7.4, T = 37 °C, 20.8 mL min^−1^) for 24 and 72 h.

Fluid shear exposure trials were performed with Fn covalently immobilized on MUA-modified 316L SS substrate with the aim of investigating the stability of the surface-attached Fn. In order to relatively quantify the effect of shear stress on the stability of Fn, ten different regions on the Fn-modified 316L SS sample surface were linearly mapped with a grazing incidence reflection (GIR) FITR. Before and after the exposure to fluid flow, the integrated intensity of the protein’s amide I vibration was determined. Additionally, for better graphical visualization and comparison, the integrated peak areas were normalized with respect to the initial (control) sample, a thoroughly cleaned 316L SS substrate modified with Fn bound via a linking MUA monolayer (Scheme 1). Measurements were performed after 24 and 72 h of continuous flow, and the obtained results are presented in Figure 4.

Although the above plot displays normalized data, the ordinate slightly extends beyond unity (100%) in the 24 h trial as some of the spectra obtained after the exposure to fluid shear lie above the initially collected spectra. However, the statistical analysis of the results presented in Figure 4 confirmed that the shear stress bore no effect on the surface amount of Fn during the 72 h of measurement, thus validating the high stability of Fn on the surface under the applied experimental conditions.

### 2.3. Investigation of the Secondary Structure of Fn Covalently Attached to the MUA Monolayer Formed on a 316L SS Surface

**Figure 5:**
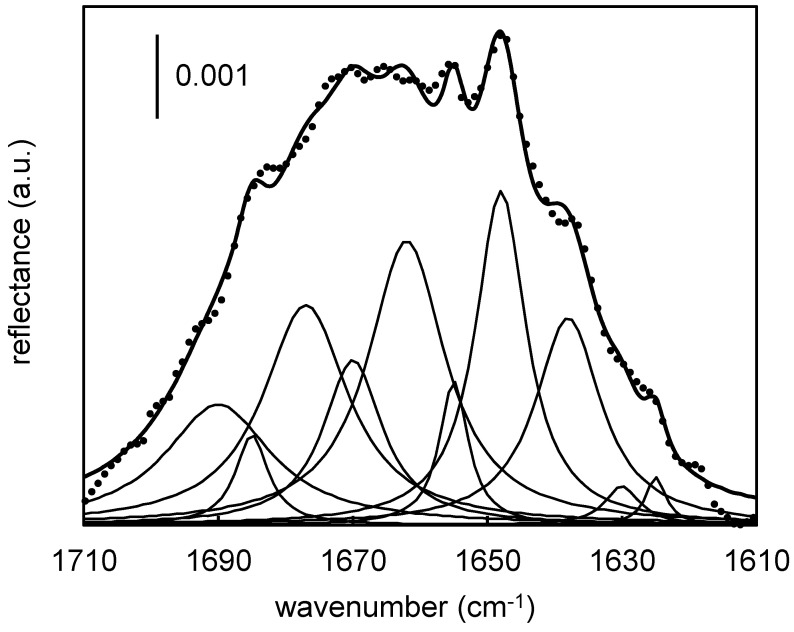
Amide I band of Fn deconvoluted into its underlying secondary structure component peaks (thin solid lines). The black dots represent the experimental PM-IRRAS spectrum of Fn chemically attached to a 316L SS surface through an MUA monolayer (Scheme 1). The thick solid curve represents the summation of the secondary structure component bands. The secondary structure bands were assigned based on the values presented in Table 1.

In this study, PM-IRRAS was used to obtain information about the secondary structure of Fn chemically immobilized on a 316L SS surface through an MUA monolayer (Scheme 1). Figure 5 shows a sample of the corresponding amide I peak deconvoluted to its secondary structure components (thin solid lines), following the assignments in Table 1 [57,58]. It can be seen that the composite simulated peak (thick solid line) fits the experimental data well. A good agreement was likewise obtained with the replicate samples.

By integrating the area under the secondary structure components’ bands (Figure 5), the proportion of each component with respect to the total area of the amide I band can be determined. Consequently, Fn covalently bound to the MUA-modified 316L SS surface (Scheme 1) was found to be composed of 34% β-sheets, 46% β-turns, 16% random coils, and 4% α-helices. The component distribution for Fn physisorbed on a bare 316L SS surface (no MUA layer) was 32% β-sheets, 40% β-turns, 23% random coils, and 5% α-helices.

By comparing the values obtained from Figure 5 to the secondary structure values of native Fn (40% β sheets, 32% β turns, 23% random coil, and 5% α helices) [59], it is evident that the covalent immobilization of Fn on the MUA monolayer formed on a 316L SS surface resulted in changes in its secondary structure. Namely, the β-sheet and random coil content decreased by 6 and 7%, respectively, whereas the β-turns and α-helix content increased by 14 and 1%, respectively. On the contrary, the Fn physisorbed on a bare unmodified surface did not show any significant changes in the content of random coils and α-helices, whereas there was an 8% increase and 8% decrease in the β-turn and β-sheet content, respectively. One possible explanation for these changes is that upon the chemical immobilization of Fn on an MUA-covered 316L SS surface, the protein molecules partially unfolded and exposed the apolar amino acid groups buried inside the protein to the surface, which consequently led to a decrease in the amount of β-sheets and random coil structures and an increase in the amount of β-turns. This indicates that Fn molecules might pose a more open conformation on an MUA-modified 316L SS surface, compared to their native structure. This relatively open conformation of Fn on the MUA-modified 316L SS surface is also in agreement with the results of our cell attachment experiment, which are to be presented in our next manuscript.

Koteliansky et al. studied the native secondary structure of Fn by circular dichroism (CD) and FTIR; their findings revealed that native Fn does not have significant α-helical structural elements, but it mainly consists of antiparallel β-forms (35%). They also concluded that the rest of the amino acids are in an unordered conformation [6]. Based on CD spectra, Osterlund et al. reported a secondary structure element distribution of 79% β-sheets and 21% β-turns for the native human plasma Fn [60]. Cheng et al. used FTIR-ATR to investigate the secondary structure of Fn (20 μg mL^−1^ in PBS, pH = 7.4) adsorbed on various SAMs. They were not able to determine the native secondary structure of Fn with ATR due to the limitations of the instrument, particularly the interference of H_2_O molecules [61]. Baujard-Lamotte et al. reported 11.5% hydrated random domains, 16% hydrated β-strands, and 28% unhydrated β-sheets for Fn molecules in 10 mM deuterated Tris buffer at pD 7.4 containing 150 mM NaCl [57]. The most recent and accurate study published by Pauthe et al. [58] revealed that Fn in its native conformation is composed of 43% β sheets, 31% β turns, and 26% unordered structures. They likewise reported no alpha-helical structure content. According to the literature, their findings are in agreement with the structure of isolated molecules deduced from X-ray and NMR measurements [62,63]. Our previous measurements of the secondary structure composition of Fn in its native state carried out using FTIR-ATR microscopy showed that Fn is composed of approximately 40% β-sheets, 32% β-turns, 23% random coil, and 5% α-helices [64]. As it is apparent from the above, different and inconclusive Fn secondary structure compositions have been reported in the literature, which might be due to different experimental methods/measurement techniques used in the research.

### 2.4. Modification of a Commercial 316L SS Coronary Stent Surface with Fn: Surface Coverage of Fn

In general, metal implant surfaces are not biologically active, i.e., they do not have active sites for cell/surface interactions. However, upon introducing an artificial biomaterial (implant) into the body, proteins from the blood and surrounding tissue adsorb onto the implant surface, making the surface biologically active (either in a positive or negative way) for further cell interaction [65,66,67]. Hence, the type and amount of the adsorbed protein determine the type, quantity, and availability of the formed biologically active sites. Moreover, the orientation and packing density of the adsorbed proteins dictate if the available active sites can trigger intracellular responses via cells’ transmembrane integrins. Although covering a biomaterial surface with Fn has been proven to have promising effects on the enhancement of the implant’s biocompatibility [22,68], the surface density of Fn is of crucial importance. Namely, if the implant’s surface remains even partially uncovered by Fn and becomes exposed to a body fluid, such as blood, incompatibility issues might arise, and the ultimate goal of surface treatment (functionalization) may become unattainable. For example, in the case of cardiovascular stents, fibrinogen may adsorb on the uncoated part of the stent’s surface and subsequently trigger blood clot formation, which is extremely dangerous for the patient [69]. Thus, it is very important to evaluate the surface coverage of Fn molecules attached to a real, commercial grade 316L SS coronary stent surface through an MUA monolayer.

Previously, most of the studies utilized immunofluorescence microscopy to visualize the distribution of Fn molecules on model flat surfaces at the microscopic level [21,70]. However, these studies mainly focused on the reorganization and conformational changes of Fn molecules in a matrix assembly or during their interactions with various cells. In the current work, immunogold labeling was used to tag Fn molecules covalently immobilized on an MUA-modified *commercial* 316L SS stent surface. For this purpose, Fn antibodies conjugated with colloidal gold nanoparticles were specifically attached to the Fn molecules immobilized on the stent surface through the primary Fn antibody and were subsequently visualized with SEM. This enabled us to verify the nano-scale distribution and surface Fn coverage on the stent.

**Figure 6:**
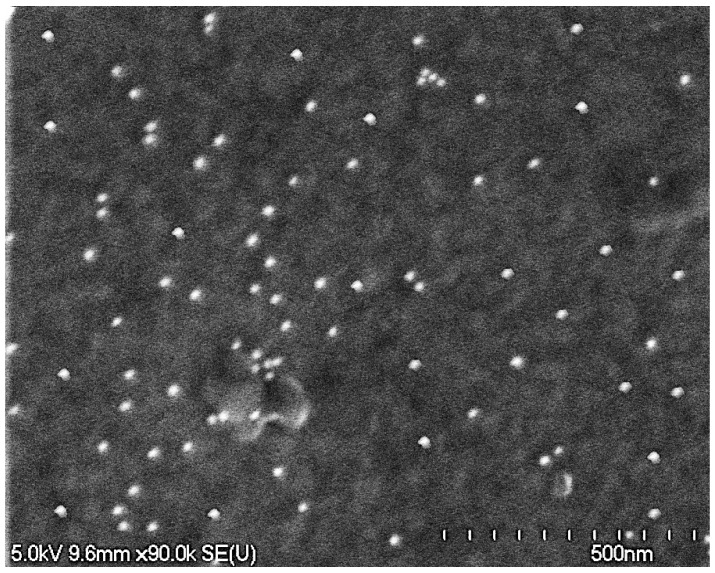
SEM image of a commercial 316L SS coronary stent surface functionalized with Fn covalently attached to the surface through an MUA monolayer and subsequently labeled with a gold conjugated antibody.

Gold nanoparticles of a smaller diameter can yield a higher labeling efficiency [71]. Therefore, in this study, 18 nm commercial gold nanoparticles conjugated to a secondary Fn antibody were used. In order to obtain higher contrast images of gold nanoparticles, a backscattered electron (BSE) detector was used in SEM imaging; however, due to the presence of various heavy elements in the underlying 316L SS alloy, even with the BSE detector, the SEM imaging was performed with an extreme effort to obtain acceptable contrast at a high magnification [72]. Figure 6 shows the resulting SEM image of an Fn/Au-functionalized commercial 316L SS coronary stent surface.

The image evidences a relatively uniform distribution of gold nanoparticles on the surface, which in turn proves the same for the Fn attached to the underlying MUA monolayer. Nevertheless, some ‘gaps’ can still be noticed. One plausible explanation for this observation could be the fact that immunolabeling is a two-step procedure: first, the primary Fn antibody was attached to Fn immobilized on the MUA monolayer, and second, the secondary antibody containing gold nanoparticles was finally attached to the primary antibody. Considering that not all of the primary antibody molecules were bound to the Fn receptor, and not all of the secondary antibody molecules were bound to the primary antibody molecules, there is a high probability that the SEM image of gold nanoparticles does not reflect the total amount of Fn molecules attached to the surface MUA monolayer.

**Figure 7:**
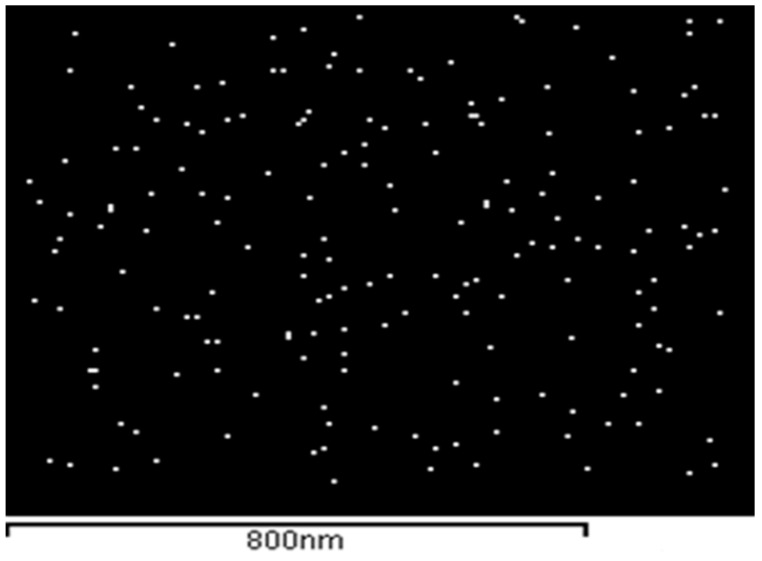
EDX mapping of the elemental gold nanoparticle distribution on a commercial 316L SS coronary stent’s surface functionalized with Fn covalently bound to it through an MUA monolayer and subsequently labeled with a gold-conjugated antibody.

In order to further verify the distribution of Fn on the surface, EDX analysis was applied to carry out gold elemental mapping. Since there was no gold in the 316L SS alloy substrate, the Au peaks were resolved to yield a high contrast spectroscopic map of gold nanoparticles on the surface, Figure 7. However, the Au_Mα1_ X-ray peak position, which is much more intensive than the Au_Lα1_, could not be used in the mapping because of its overlap with the S_Kα1_ peak of the thiol groups of the MUA; therefore, the Au_Lα1_ line was used instead.

Figure 7 shows a more homogeneous and denser distribution of gold nanoparticles on the surface than that in Figure 6 (note the difference in the actual lengths of the scale-bars on the two images). The resulting X-ray map clearly illustrates that there is a uniform coverage of Fn molecules on the stent surface. Moreover, on the control samples (bare metal stents), no signs of the presence of gold particles in SEM images and EDX maps were detected, evidencing that the gold-labeled antibody exclusively confirmed the presence of Fn on the stent surface. A nearly similar distribution of gold particles was observed on the inner and outer surfaces of the stent, and the ensuing SEM/EDX analysis of multiple sections of the stent did not reveal any intact (non-Fn-modified) spots. This implies that the MUA surface modification and Fn immobilization techniques enabled efficient modification of the entire surface of the commercial stent. It should be mentioned that no gold particles were observed on the control surfaces (bare and MUA-modified 316L SS stent), which likewise indicates that the affinity of antibodies to surfaces in the absence of Fn is extremely low.

As mentioned earlier, cells interact with ECM proteins via their transmembrane receptors, integrins [66]. After the cells bind to a specific adhesive ECM ligand, such as RGD (an arginine–glycine–aspartic amino acid sequence), several intracellular anchor proteins are clustered at cell–ECM adhesion sites. This eventually leads to the assembly of focal adhesions (FAs), which connect the cell’s cytoskeleton to the ECM proteins [73]. It is widely accepted that cells can detect and respond to the physicochemical features of the surface to which they adhere. Among them, micro- and nano-topography, the chemical nature of the surface, and the structure and spacing of ECM proteins are well documented [74,75,76,77]. In addition, numerous studies revealed that the spacing between binding motifs on ECM proteins significantly affects cell adhesion and intracellular signaling [76]. Particularly, for effective FAs, the optimal ligand spacing is reported to be between 60 and 140 nm. For example, Massia and Hubbel immobilized RGD peptides on glass and investigated the effect of RGD spacing on the spreading of human foreskin fibroblasts (HFFs) and the formation of focal contact and stress fibers [78]. They demonstrated that a minimum 440 nm RGD spacing is sufficient for HFF spreading; however, FA was not actively formed at this spacing. Their findings revealed that optimum focal contact and stress fibers can only form at high RGD surface densities, where the spacing is approximately 140 nm. Cavalcanti-Adam et al. investigated the effect of 58 and 108 nm RGD spacing on rat fibroblasts. They reported that cells plated on a 108 nm space pattern showed a delayed spreading compared to the cells plated on a 58 nm space pattern. In addition, they showed that the cells cultured on the 58 nm space pattern formed normal FAs, whereas on the 108 nm space pattern, FAs were not assembled. In another study, Hughes et al. coated a plastic substrate with hamster plasma Fn and reported a minimum of 1.8 × 10^10^ Fn molecules per cm^2^ surface density (approximately 74 nm spacing) for maximal spreading of baby hamster kidney (BHK) fibroblasts [79].

In our study, the image analysis of the EDX elemental map (Figure 7) revealed an average surface density of 2.3 × 10^10^ Fn molecules per cm^2^, and if we assume that the particles were distributed uniformly, the Fn center-to-center molecular spacing on the stent surface can be estimated to be approximately 66 nm. Based on our previous experiments [26], this indicates that most of the Fn molecules are in a closed-like conformation. Namely, Dargahi et al. determined that an average diameter of Fn adsorbed in a closed conformation is 45 nm, while that adsorbed in an extended conformation is 142 nm [26]. Thus, the average 66 nm spacing in Figure 7 falls within the reported optimum range of 60 to 140 nm [75,76] and indicates that an Fn-modified 316L SS stent surface makes a very suitable substrate for the formation of FAs and good cell spreading. Particularly for ECs, Le Saux et al. reported that the optimal RGD spacing that favors the adhesion of bovine aortic ECs and the formation of FAs is about 44 nm, which is agreeably close to our findings [76]. However, it should be noted that in all of the aforementioned studies, due to the nature of the experiments, certain discrete ligand spacings were evaluated and optimum values were reported. For example, in the study conducted by Le Saux et al., 1.4, 44, 138, 1380, and 43,700 nm RGD spacings were examined and the optimum value of 44 nm was reported [76]. No other RGD densities were examined within the range spanning these values. Hence, the 66 nm spacing of Fn molecules obtained in this study may likewise be effective enough to enhance EC adhesion and FA formation. In our upcoming manuscript, this will, indeed, be demonstrated to be the case.

## 3. Material and Methods

### 3.1. Immobilization of Fn on MUA-Modified 316L SS, NiTi, and L605 CoCr Surfaces

An 11-mercaptoundecanoic acid (MUA) monolayer was electrochemically formed on the surface of 316L SS (McMaster-Carr 9298K131), L605 CoCr (HAYNES^®^ alloy 25-L605-UNS R30605), and NiTi (Alfa Aesar, wt% Ni 55.82, Ti balance, C ≤ 0.05, O ≤ 0.05, total of all other metal impurities ≤ 0.20) following the protocol presented in a previous paper [49]. After the MUA monolayer formation, the samples were sonicated in deionized water and denatured alcohol for 10 min in order to remove any physisorbed MUA molecules from the surface. Following sonication, the samples were immersed in an aqueous solution of phosphoric acid (pH 1.5) for 30 min in order to eliminate the sodium-carboxylate complex formed with the MUA monolayer and to make the MUA chemically active (Scheme 1). The samples were rinsed again with deionized water to remove any residual phosphoric acid from the surface. At this stage, a conventional protocol was followed to covalently attach Fn to the underlying MUA monolayer (Scheme 1).

In brief, the samples were immersed in an aqueous solution of 15 mM n-hydroxysuccinimide (NHS) and 75 mM 1-ethyl-3-(3-dimethylaminopropyl) carbodiimide (EDC) for 2 h to form a succinimidyl ester intermediate on top of the monolayer. The samples were then rinsed with deionized water and immediately exposed to a 40 µg mL^−1^ Fn solution in phosphate buffer (pH = 6 and T = 4 °C) for 12 h in order to react the free amine groups of the Fn molecules with the ester groups of NHS on the sample’s surface (Scheme 1). This reaction leads to the replacement of the NHS groups on the surface and the formation of covalent amide bonds between Fn molecules and the MUA monolayer on the surface. The Fn-modified samples were then sonicated in 0.1 M NaOH for 10 min to hydrolyze the unreacted ester groups as well as to remove any loosely physisorbed Fn molecules by decreasing the electrostatic interactions between Fn and the surface.

### 3.2. Polarization Modulation Infrared Reflection Adsorption Spectroscopy (PM-IRRAS)

A Fourier-transform infrared (FTIR) spectrometer equipped with an external polarization modulation module was employed to record the PM-IRRAS spectra. The instrument was equipped with a mercury cadmium telluride (MCT) detector, which was cooled with liquid nitrogen. In order to acquire the highest reflectance amplitude, the polarized IR beam was emitted at the samples at an 80° angle to the surface normal. The spectra were averaged over 128 scans at 3 cm^−1^ resolution.

### 3.3. Parallel Plate Flow Chamber Experiments

The same experimental procedure used to investigate the impact of shear stress on the MUA layer stability [49] was also applied to investigate the impact of shear stress on the Fn monolayer stability. It is worth noting that the infrared (IR) integrated intensity of the amide peak of Fn was used as a correlation metric for surface coverage instead of the methylene peaks of MUA in the earlier study. All freshly prepared Fn-modified surfaces were first sonicated in 0.1 M NaOH in order to prevent erroneous high coverage readings caused by loosely physisorbed protein molecules. Similarly, a background scan was collected with a blank, mirror-polished 316L SS or CoCr or NiTi sample, which was first rinsed with deionized water, degreased with ethanol, and thoroughly cleaned in an ultrasonic bath before the measurement.

### 3.4. Determination of Fn Secondary Structure

An IR spectrum of proteins adsorbed on a surface carries information about the amount and the secondary structure of the protein layer. PM-IRRAS is a convenient technique that can be used to evaluate the secondary structure elements of proteins. It is a non-invasive and surface-sensitive technique producing IR spectra that are highly sensitive to the secondary-structure changes of adsorbed proteins.

Two major broad bands near 1610–1710 cm^−1^ (amide I) and 1500–1600 cm^−1^ (amide II) are commonly attributed to proteins in the mid-IR region. The exact wavenumber and shape of these peaks are highly dependent on the secondary structure of the protein. The amide I band represents carbonyl peptide stretching modes, whereas the amide II band corresponds to in-plane N-H bending vibration modes in proteins. Amide II is not particularly responsive to conformational changes; on the other hand, all of the secondary structural elements including α-helix, β-sheet, β-turn, and random coil structures raise vibrations in the 1600 to 1700 cm^−1^ region. For this reason, the amide I band is used primarily to study the secondary structure and adsorption coverage of proteins on surfaces.

Second derivative analysis was carried out with the recorded amide I spectra to estimate the number of secondary structure components of Fn bound to the MUA monolayer on a 316L SS surface and determine their corresponding IR peak positions. As discussed earlier, the size and position of secondary structure elements depend on the type of protein and its conformation. Hence, using the literature information, certain peaks were chosen, and the components were assigned to the peaks obtained from the second derivative of the amide I peak (Table 1) [57,58]. By referring to the values presented in Table 1, the experimental amide I band was fitted to the secondary structure components using a curve-fitting function in conjunction with the iterative least-squares Levenberg–Marquardt algorithm. The peak positions as well as the type of curve (Gaussian) were fixed, while the width and height of each component band were iterated in order to obtain the best fit between the simulated and experimental amide I peak. All the calculations were performed in OPUS 5.5 software provided by Bruker Optics.

### 3.5. Immunolabeling of Fn with Colloidal Gold Nanoparticles

After covalently binding Fn to an MUA-modified commercial 316L SS stent, the stent was incubated in a blocking solution containing 5% BSA (*g*/*v*) in PBS (pH = 6) for 2 h at 4 °C. After that, the immobilized Fn molecules on the surface of the stent were reacted with a primary antibody (1/50 (*v*/*v*) diluted rabbit anti-Fn polyclonal antibody from Millipore, CAT = AB2047) for 1 h to recognize the presence of covalently bound Fn on the MUA-modified surface. The stent was then washed and blocked once more as explained above, followed by a reaction with a gold-labeled secondary antibody (1/50 (*v*/*v*) diluted donkey anti-rabbit IgG 18 nm gold, Jackson ImmunoResearch Laboratories Inc. #711-215-152) for 30 min. According to the manufacturer, the secondary antibody was diluted in 0.5 M NaCl buffered in 0.1% (*g*/*v*) BSA, 0.05% (*v*/*v*) TWEEN^®^ 20, and 5% (*g*/*v*) fetal bovine serum (pH = 6) to minimize background staining. The stent was then washed repeatedly in PBS and once with deionized water. Finally, the stent was immersed in a 1% (*v*/*v*) glutaraldehyde fixation solution to stabilize the antigen–antibody–gold complex on the surface. Three sets of control samples were used to verify the data: a naked 316L SS, an MUA-modified 316L SS, and an Fn-modified 316L SS surface that had not undergone primary antibody fixation. For visualization, the samples were mounted onto metallic SEM stubs with conductive carbon cement. The conjugated gold nanoparticles were then imaged by a field-emission gun scanning electron microscope, FE-SEM, which was operated at an accelerating voltage of 5 kV and a working distance of <10 mm. The stent samples were oriented to provide a full visual survey of the inner, outer, and transverse cross-section of the stent in order to assess the distribution of Fn on the whole surface of the stent. In addition to imaging, elemental mapping of the stent surface was performed using the energy dispersive X-ray (EDX) method.

## 4. Conclusions

In this study, an attempt was made to immobilize Fn to 316L SS, CoCr, and NiTi flat samples and on the commercial 316L SS coronary stent. The recorded PM-IRRAS spectra corroborated the presence of covalently bound Fn molecules on these surfaces. Simulated blood flow shear stress experiments revealed that the resulting Fn layer was very stable on the 316L SS surface. It was shown that the immobilization of Fn on a 316L SS surface causes significant changes in its secondary structure. Finally, the SEM and EDX analyses of the Fn molecules immobilized on the surface of a commercial 316L SS cardiovascular stent revealed that a relatively homogenous surface coverage of Fn was achieved, and the molecular spacing was appropriate for cell adhesion and FA formation. These findings prove that the covalent binding method used with the three investigated alloys was efficient in functionalizing their surfaces with Fn. However, it should be noted that a range of other functional molecules could be used in place of Fn, including but not limited to immunoglobulins, DNAs, antibodies and other proteins, drug molecules, etc.

## Figures and Tables

**Table 1:** Peak positions assigned to secondary structure components in Fn covalently bonded on 316L SS surface through MUA SAM.

Secondary Structure Element	Peak Position (cm^−1^)
β sheet	1618
β sheet	1625
β sheet	1630
β sheet	1638
random coil	1648
α helix	1655
β turn	1662
β turn	1670
β sheet	1677
β turn	1685
β turn	1690

## Data Availability

Data are contained within the article.

## Supplementary Materials

The following supporting information can be downloaded at: https://www.mdpi.com/article/10.3390/molecules29204927/s1, Figure S1: PM-IRASS responses of a modified (a) CoCr and (b) NiTi surface. The dotted line represents the response of an NHS activated surface, whereas the solid line represents that of an Fn-modified surface.
