# Supplementary material for: Modification of 316L Stainless Steel, Nickel Titanium, and Cobalt Chromium Surfaces by Irreversible Immobilization of Fibronectin: Towards Improving the Coronary Stent Biocompatibility
Source: Molecules. 2024 Oct 18;29(20):4927. doi: 10.3390/molecules29204927 (PMC11510294; doi:10.3390/molecules29204927)
Supplement: Supplementary file 1 [file molecules-29-04927-s001.zip › molecules-3245182-supplementary.pdf]

Modification of 316L Stainless Steel, Nickel Titanium and Cobalt Chromium Surfaces by Irreversible Immobilization of Fibronectin: Towards Improving the Coronary Stent Biocompatibility

Hesam Dadafarin [1], Evgeny Konkov [1], Hojatollah Vali [2], Irshad Ali [3] and Sasha Omanovic [1,*]

[1] Department of Chemical Engineering;, McGill University, 3610 University St., Montreal, QC, H3A 0C5, Canada hesam.dadafarin@mail.mcgill.ca (H.D.); evgeny.konkov@mail.mcgill.ca (E.K.)
[2] Department of Anatomy and Cell Biology, McGill University, 3640 University St., Montreal, QC, H3A 0C7, Canada; hojatollah.vali@mcgill.ca (H.V.)
[3] Department of Chemical Engineering, Faculty of Mechanical, Chemical and Industrial Engineering, University of Engineering & Technology, Jamrud Road, Peshawar, Pakistan; irshad.ali@mail.mcgill.ca (I.A.)
* Correspondence: sasha.omanovic@mcgill.ca (S.O.)

SUPPLEMENTARY MATERIAL

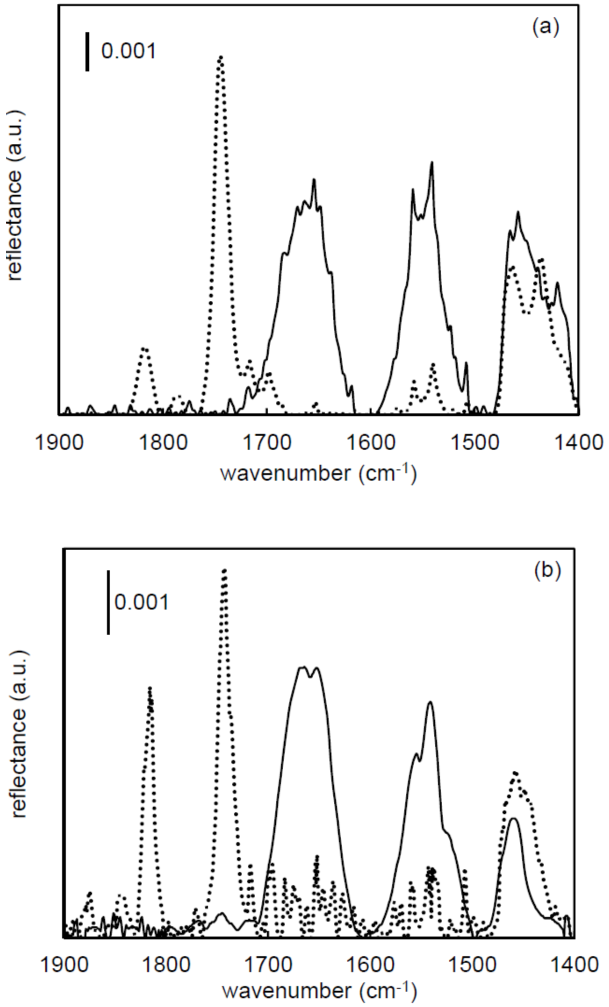

Figure S1. PM-IRASS responses of a modified (a) CoCr and (b) NiTi surface. The dotted line represents the response of an NHS activated surface, whereas the solid line represents that of an Fn-modified surface.
